# Spectrum of Female Hypospadias: A Case Series

**DOI:** 10.7759/cureus.71703

**Published:** 2024-10-17

**Authors:** Sreelakshmi Madhusoodanan, Aravind C Soman, Hridya Menon

**Affiliations:** 1 Pediatric Surgery, Government Medical College, Kozhikode, Kozhikode, IND

**Keywords:** female hypospadias, genitourinary anomaly, micturating cystourethrogram, mitrofanoff procedure, neurogenic bladder

## Abstract

Female hypospadias is an extremely rare condition characterised by urethral meatus placed within the introitus on the anterior vaginal wall, along with foreshortening of the urethra. It is an often missed anomaly that usually co-exists with other genitourinary conditions like ectopic ureter, renal anomalies, septate vagina and bicornuate uterus.

A case series of five girls who had hypospadias is being described. All the children had an early onset of recurrent episodes of urinary tract infections (UTIs). All of them had voiding dysfunction in the form of dribbling, straining or obstruction to their urinary outflow. On cystoscopic evaluation, all of them had trabeculated bladder, open bladder neck and poorly formed trigone, with the function of at least one kidney compromised on nuclear scintigraphy. Two children in the series had isolated female hypospadias with backpressure changes in the bladder. Two girls had associated anterior ectopic anus, and one girl had a septate vagina with a bicornuate uterus, suggesting abnormal urogenital sinus development. One child had a spinal abnormality, which would have contributed to the neurogenic bladder.

Female children with recurrent UTIs and voiding problems should be carefully evaluated for primary urethral and bladder abnormalities. Children diagnosed with hypospadias-related neurogenic bladder have a high risk of progressing to renal failure at an early age and need bladder drainage procedures to prevent the same.

## Introduction

Female hypospadias is characterised by abnormal ventral placement of the urethral meatus along with shortening of the urethra. In contrast to male hypospadias, female hypospadias is an extremely rare and often missed anomaly that usually co-exists with other genitourinary conditions. There is very minimal existing literature regarding this entity [1,2]. There are currently no definite protocols or guidelines for the management of the same.

Diagnosis in children is usually made when evaluated for associated anomalies, urinary tract infections (UTIs) or incontinence [3,4]. Management of the condition requires an individualised approach based on the position of the opening, detailed evaluation of bladder and upper tracts, and associated malformations.

A retrospective review of the case files of five female hypospadias cases admitted to our department from June 2014 to June 2024 was conducted. All the girls included in the study had a normal-looking introitus and posterior fourchette, with the urethral meatus located in the anterior wall of the lower one-third of the vagina. Clinical presentation, investigations, management and post-operative outcomes of the children were studied.

## Case presentation

Case 1

**Figure 1:**
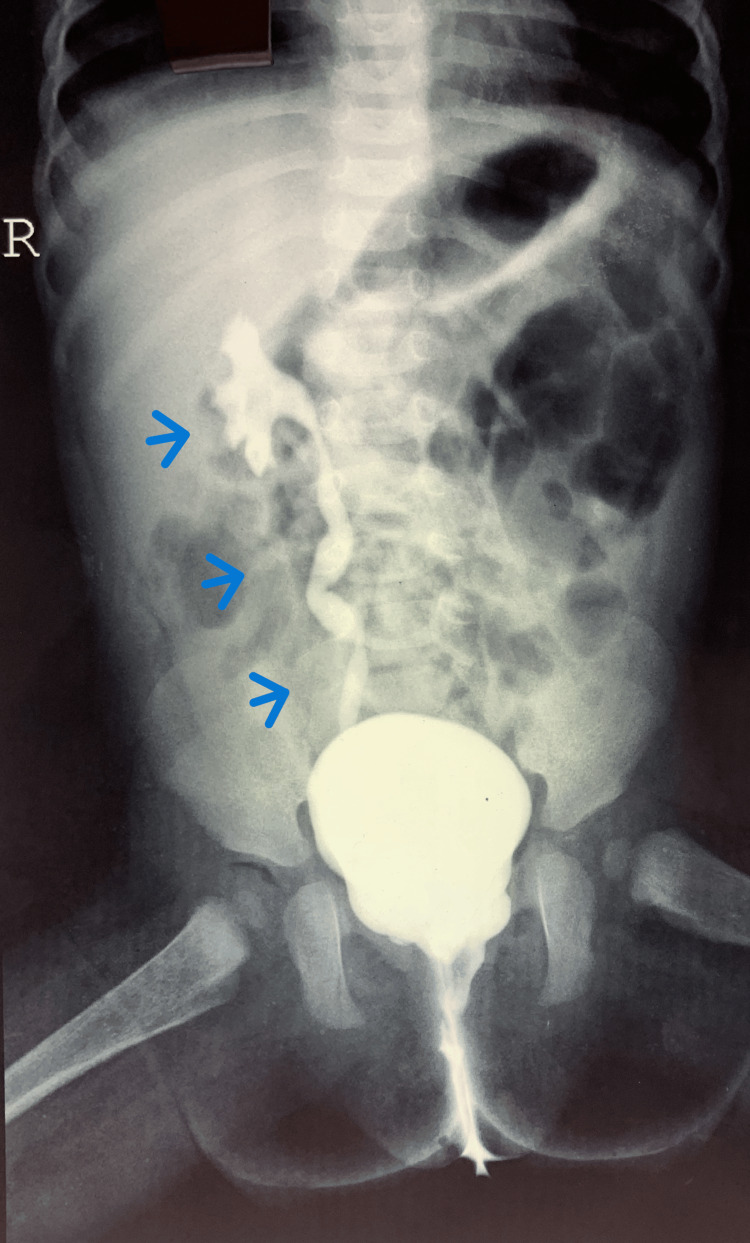
Micturating cystourethrogram (MCU) showing right grade 3 vesicoureteric reflux (VUR). The blue arrows indicate the right refluxing kidney.

**Figure 2:**
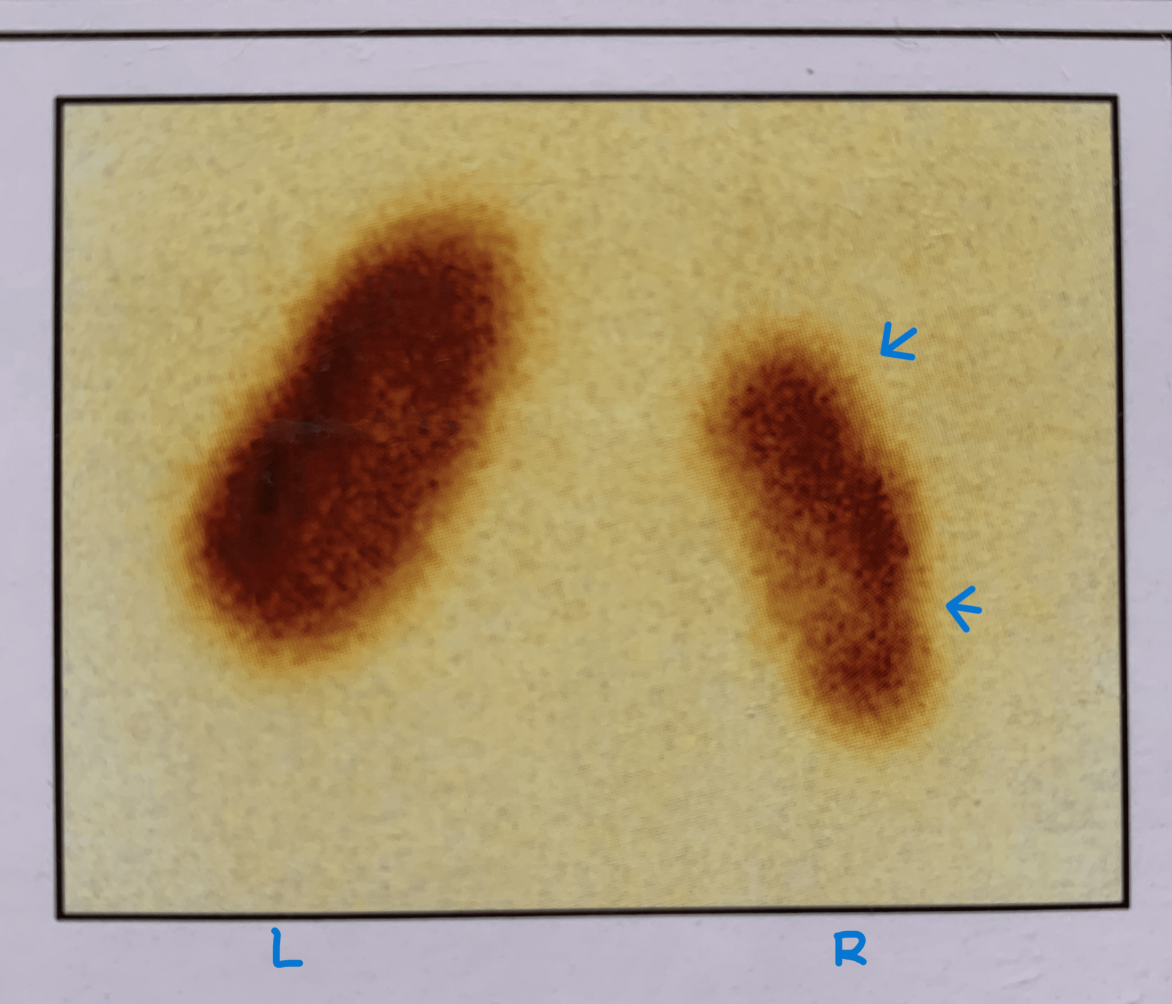
Nuclear renogram (DMSA) showing right renal scarring with decreased function (relative function of the right kidney: 35%). The blue arrows point to the scars. DMSA: dimercaptosuccinic acid

A three-year-old girl presented with a one-year history of recurrent UTIs. She also had an increased frequency of micturition and occasional urinary dribbling. Her ultrasound (US) reported right mild hydronephrosis, a bladder capacity of 110mL with a post-void residual (PVR) urine of 20mL. A micturating cystourethrogram (MCU) was done and it showed right grade 3 vesicoureteric reflux (VUR) (Figure 1) with significant PVR. Thirty-five per cent relative function and scarring of the right kidney was seen on nuclear scintigraphy (dimercaptosuccinic acid, or DMSA) (Figure 2). She underwent a cystoscopy for further evaluation that revealed a hypospadiac urethral meatus, 0.5cm proximal to the introitus, trabeculated bladder filled with flakes and patulous right ureteric orifice. Her magnetic resonance imaging (MRI) spine was normal. The child was initially given oxybutynin, tamsulosin and antibiotic prophylaxis. However, she continued to have frequent infections. Hence, she was taken up for a Mitrofanoff procedure with right ureteric reimplantation. The child is UTI-free on follow-up.

Case 2

**Figure 3:**
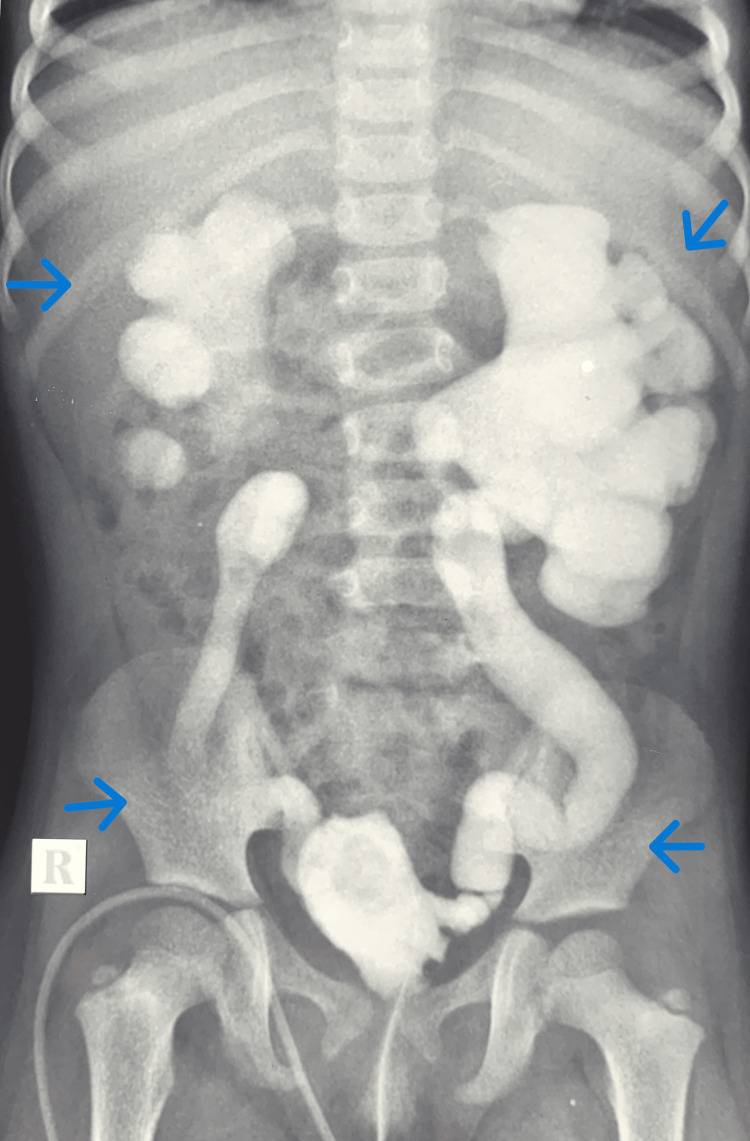
Micturating cystourethrogram (MCU) showing bilateral grade 5 vesicoureteric reflux (VUR). The blue arrows indicate the grade 5 VUR.

**Figure 4:**
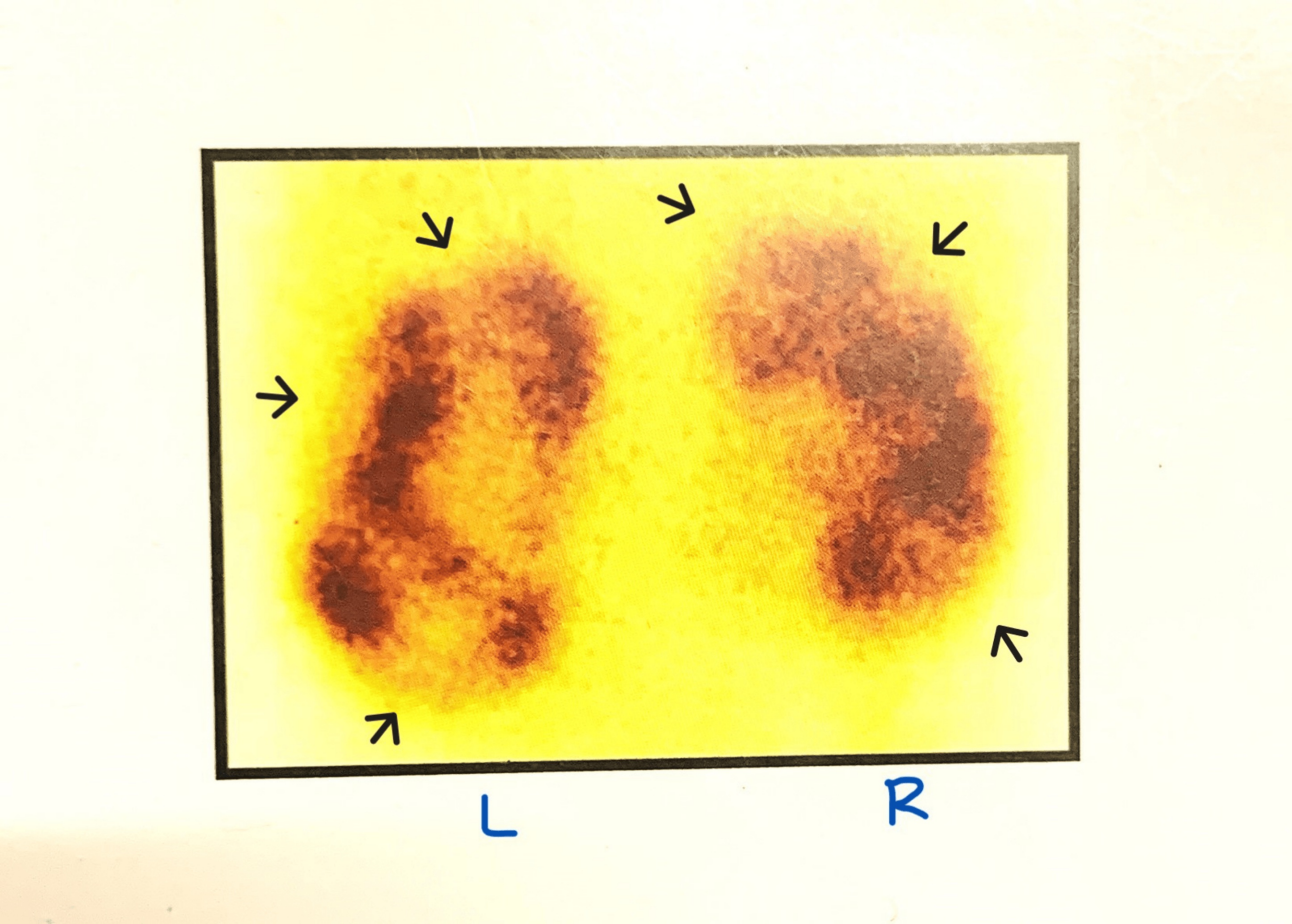
Nuclear renogram (DMSA) showing bilateral renal scarring. The black arrows indicate photopenic areas suggestive of scars. DMSA: dimercaptosuccinic acid

A two-year-old female child was evaluated for frequent urinary dribbling and febrile UTI. She had a raised serum creatinine of 2.5mg/dL at presentation. She had anterior ectopic anus that was treated with anterior sagittal anorectoplasty previously. On clinical examination, she had a palpable bladder, and her urethra was found to be hypospadiac on attempted catheterisation. US abdomen showed bilateral gross hydroureteronephrosis with a bladder wall thickness of 4mm and PVR of 64mL. Her MCU was suggestive of bilateral grade 5 VUR (Figure 3) and her DMSA showed bilateral renal scarring (Figure 4). MRI of lumbosacral spine was taken and was reported to be normal. Cystoscopy done under general anaesthesia revealed a hypospadiac urethral meatus just proximal to the introitus, short urethra with open bladder neck, trabeculated bladder with bilateral golf hole orifices and poorly formed trigone. The child underwent a Mitrofanoff procedure with bilateral ureteric reimplantation as her bladder capacity seemed adequate intra-operatively. Her serum creatinine came down to 0.5mg/dL on follow-up and she is remaining infection-free on clean intermittent catheterisation (CIC).

Case 3

**Figure 5:**
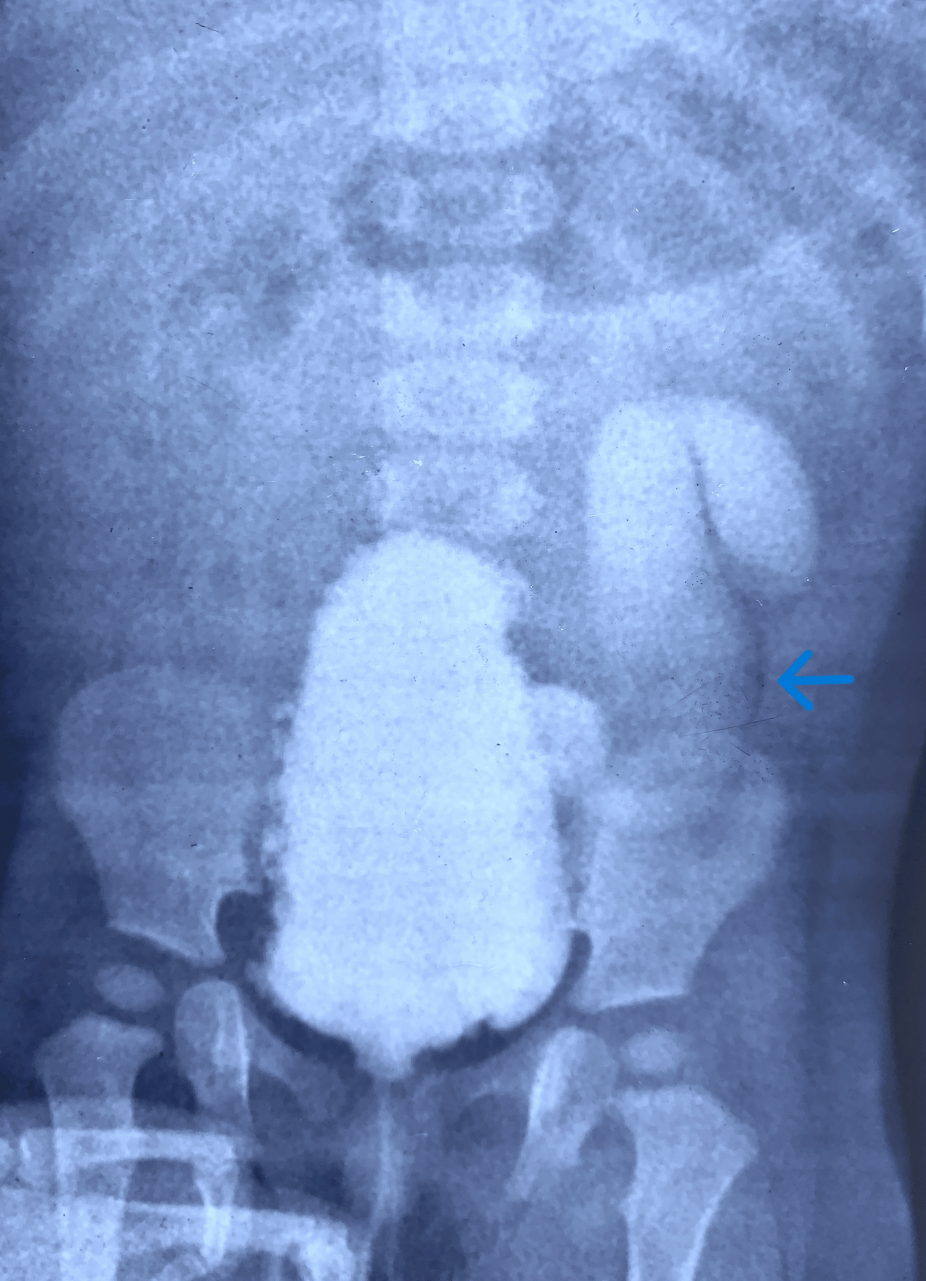
Micturating cystourethrogram (MCU) showing neurogenic bladder with left grade 5 vesicoureteric reflux (VUR). The blue arrow indicates the refluxing ureter.

**Figure 6:**
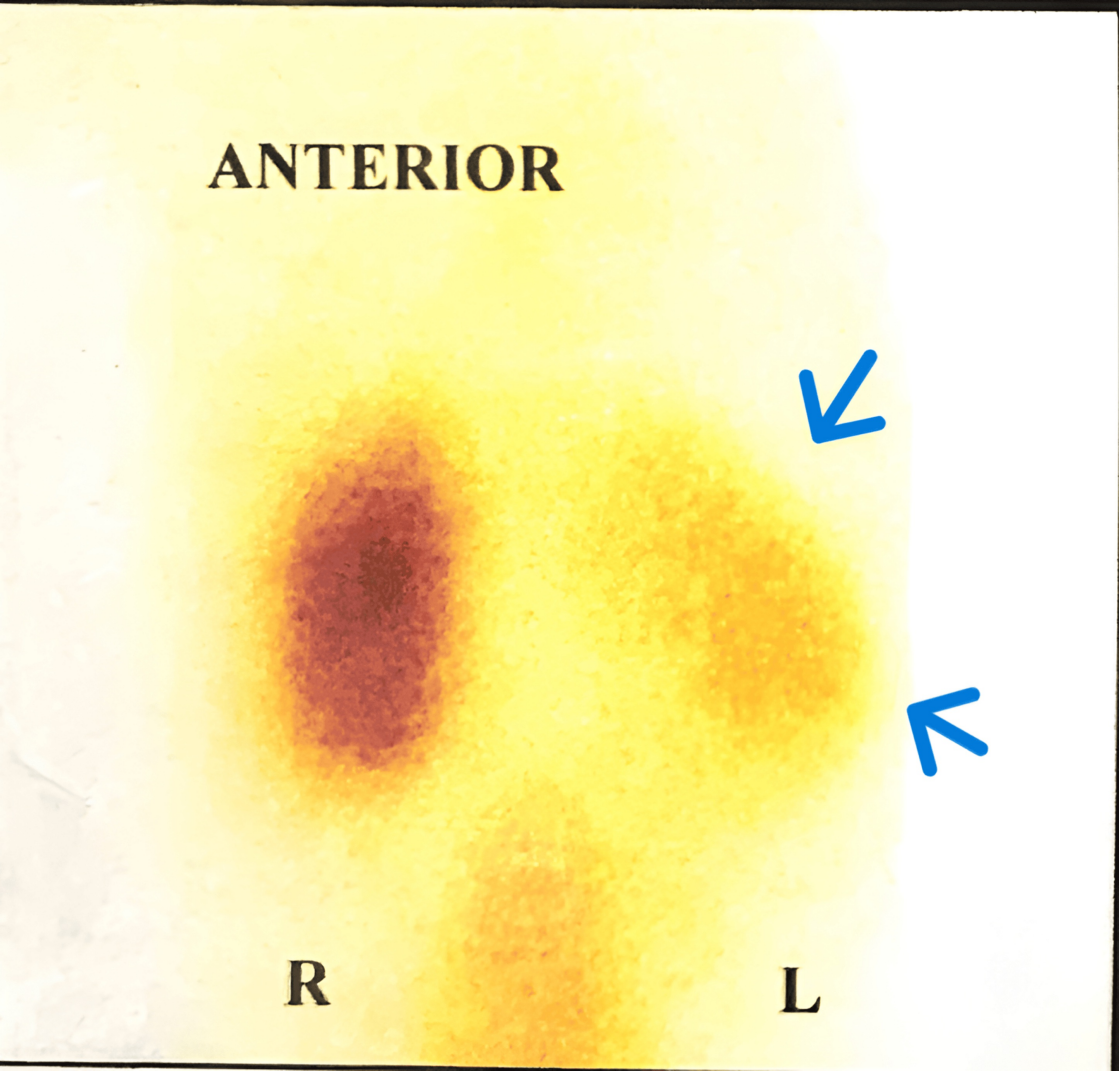
Nuclear renogram (DMSA) showing poorly functioning left kidney with 25% relative function. The blue arrows indicate the poorly functioning left kidney. DMSA: dimercaptosuccinic acid

A girl aged five years presented to casualty with acute urinary retention. Her mother gave a history of straining while voiding with increased urinary frequency since infancy. On clinical examination, she had a bladder distended up to the umbilicus; however, catheterisation failed as the urethra was not visualised. She underwent a US that showed bilateral hydroureteronephrosis and an overdistended bladder with wall thickening. Her serum creatinine was 2.3mg/dL. The child was catheterised under anaesthesia as her initial attempts at catheterisation in the emergency department had failed. Cystoscopy done in the same sitting revealed a urethral orifice within the anterior vaginal wall, 1cm proximal to introitus, with short posteriorly unsupported urethra, poorly formed bladder neck and trigone, and trabeculated bladder with sacculations. Left patulous and right slightly dilated orthotopic ureteric orifices were seen. An MCU was taken after the procedure which showed a trabeculated and elongated bladder with grade 5 VUR on the left side (Figure 5). The left kidney was scarred with 25% relative function on DMSA (Figure 6). MRI spine taken was normal. The child is on continuous bladder drainage at present as CIC was not feasible and her creatinine is 0.8mg/dL. She is planned for Mitrofanoff with left ureteric reimplantation.

Case 4

**Figure 7:**
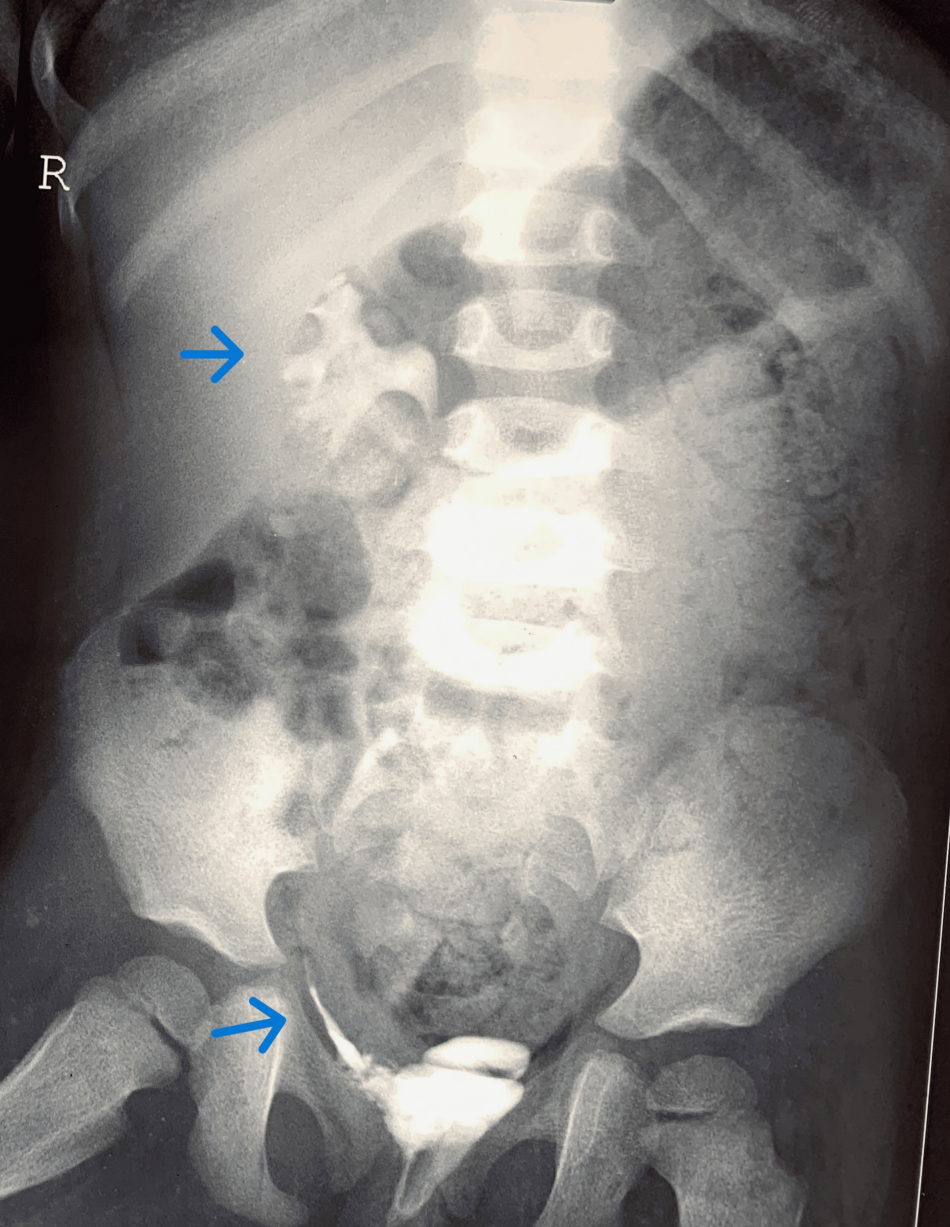
Micturating cystourethrogram (MCU) showing right grade 3 vesicoureteric reflux (VUR). The blue arrows point to the refluxing right kidney and ureter.

**Figure 8:**
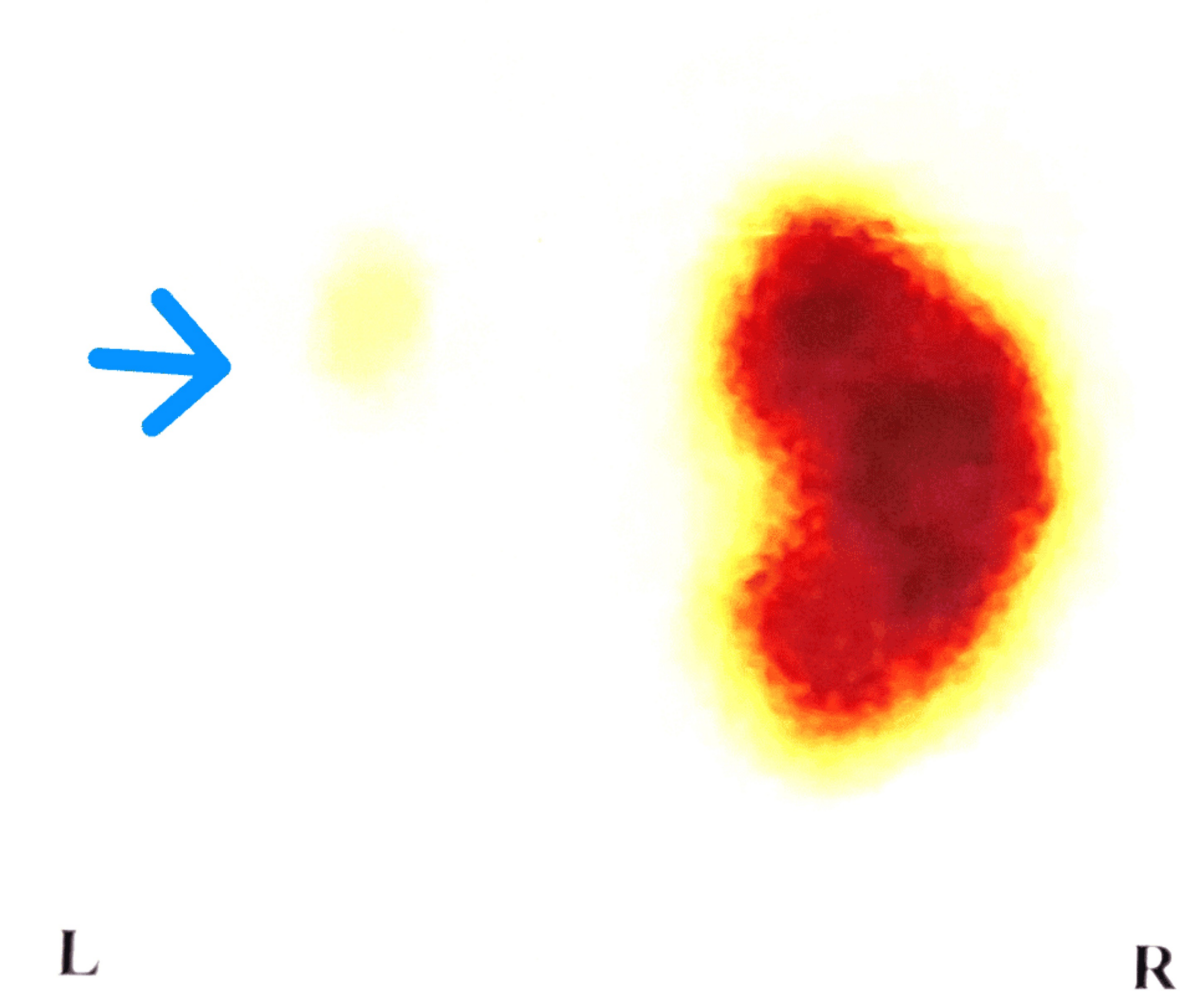
Nuclear renogram (DMSA) showing a poorly functioning left kidney. The blue arrow points to the negligible left renal uptake (2% relative function). DMSA: dimercaptosuccinic acid

A seven-year-old girl presented to outpatient with recurrent episodes of febrile UTI from birth. She also had a history of continuous urinary dribbling from infancy. The US was done for the child that showed a right normal kidney with moderate hydroureteronephrosis and a left small, contracted kidney. MCU was done that demonstrated a small capacity bladder with right grade 3 VUR (Figure 7). DMSA showed negligible left renal function (Figure 8). She also underwent MRI that reported a short urethra, incomplete septate vagina and bicornuate bicollis with a rudimentary uterus and cervix, and normal lumbosacral spine. The child was taken up for a subsequent cystogenitoscopy that revealed a urethral opening 1cm proximal to the introitus, short urethra, small capacity bladder with open bladder neck and ill-defined trigone. The right ureteric orifice was patulous and laterally placed, and the left orifice was non-visualised. The oblique septum was seen dividing the vagina into anterior and posterior compartments with the uterine cervix noted in the posterior compartment. Her serum creatinine progressively increased from 0.5mg/dL at 11 months of age to 2.3mg/dL at seven years. She is being planned for bladder augmentation with the Mitrofanoff procedure.

Case 5

**Figure 9:**
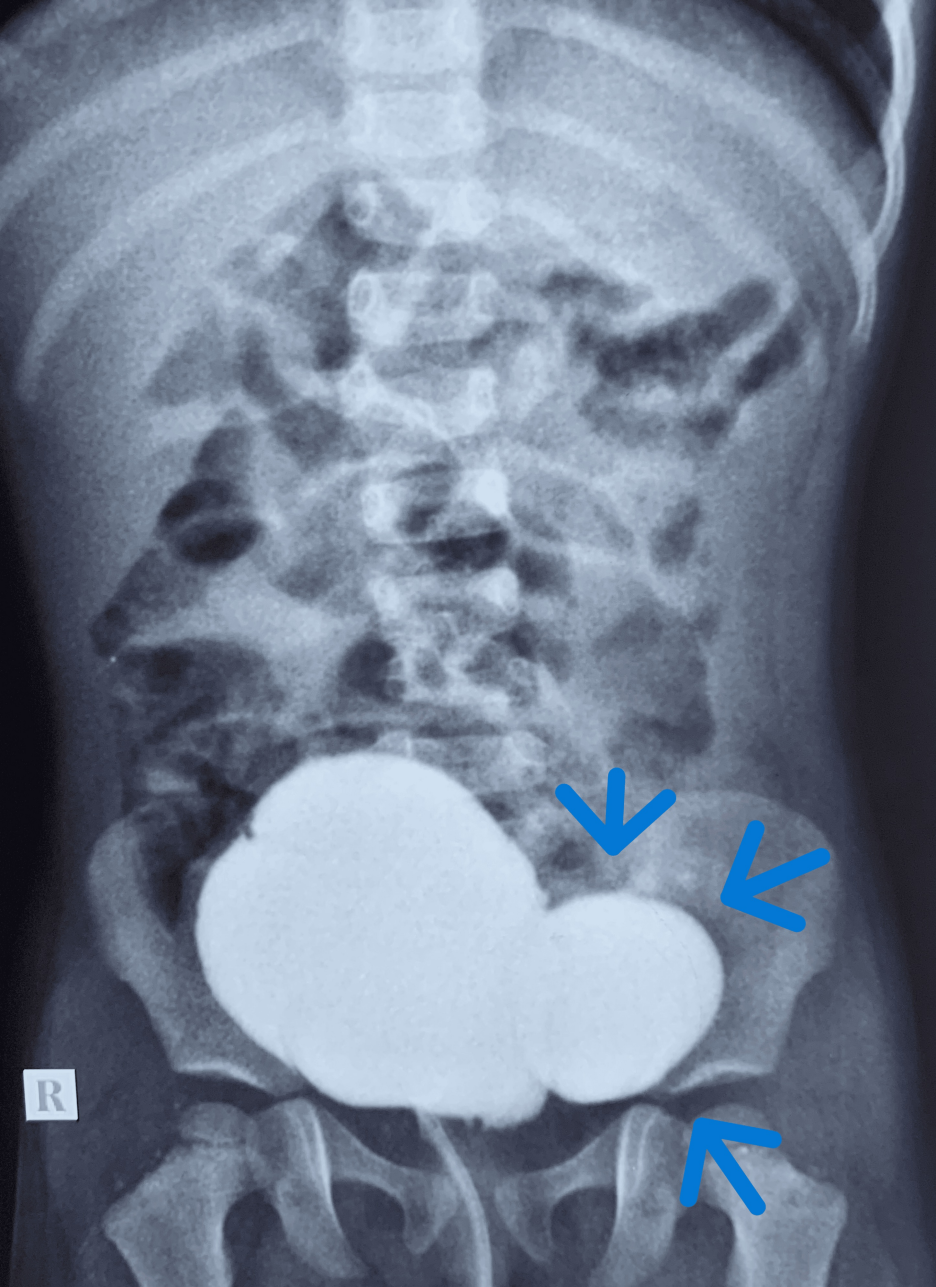
Large paraureteric diverticulum demonstrated on micturating cystourethrogram (MCU) (blue arrows).​

**Figure 10:**
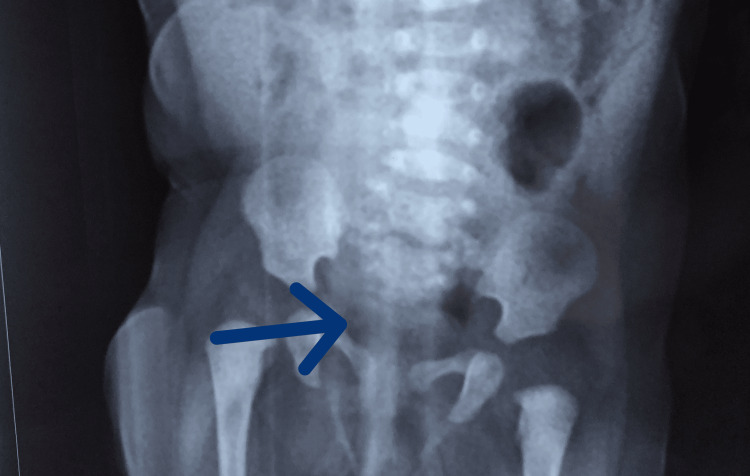
X-ray showing partial agenesis of the fourth and fifth sacral vertebrae. The blue arrow points to the sacrum.

**Figure 11:**
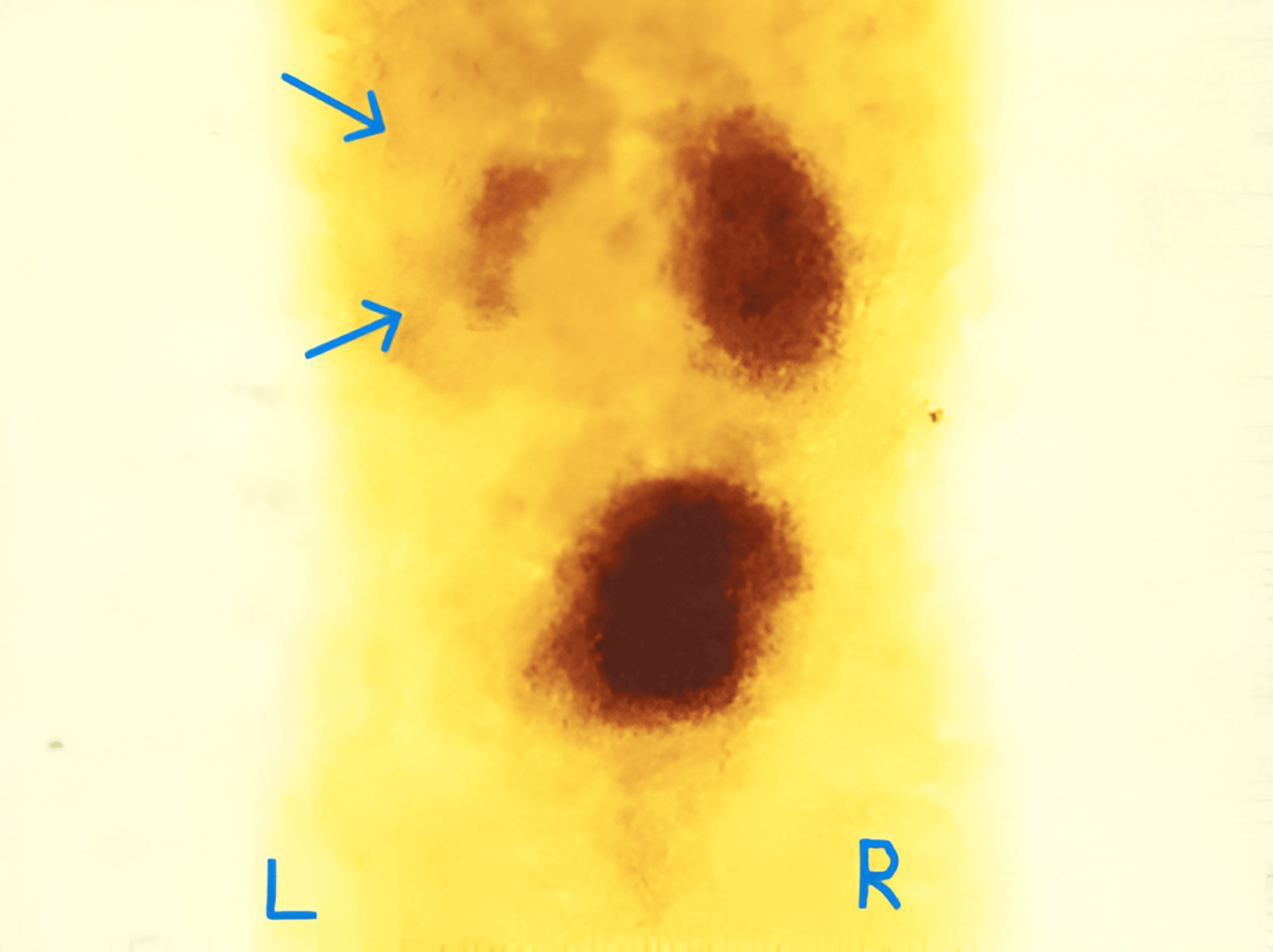
Diethyl triamine penta-acetic acid (DTPA) renogram showing poor function of the left kidney (15% relative function). The blue arrows point to the poorly functioning left kidney.

A child presented at one year of age to the paediatric surgery outpatient department with recurrent episodes of febrile UTI. She also had a poor urinary stream with excessive straining on micturition. Examination of her perineum revealed clitoromegaly and an anterior ectopic anus. The US of the perineum showed a 2.9cm x 2.5cm lesion around the left anterolateral wall of the vagina with subcutaneous extension. Gross left hydroureteronephrosis with left paraureteric diverticulum was noted on the abdominal US. Her MCU revealed a large left-sided bladder diverticulum with absent VUR (Figure 9). She also had partial sacral agenesis on her X-ray evaluation (Figure 10). An MRI spine was taken that showed partial sacral agenesis with absent cord tethering. On cystogenitoscopy, her urethral opening was seen on the anterior vaginal wall, 1cm proximal to the introitus. It had an associated cystic structure on its dorsal surface, extending into the corpus spongiosum, with an opening in the clitoris, suggestive of a duplicated urethra. The short ventral urethra entered a trabeculated bladder filled with flakes. A large left paraureteric diverticulum was seen. The child underwent an excision of the cystic dorsal urethra followed by anterior sagittal anorectoplasty. Her urinary stream improved after the procedure. A left nephroureterectomy was done two years later for deteriorating left renal function (from 15% to 5% relative function on nuclear scintigraphy) (Figure 11) and recurrent infections. The child is currently under follow-up and has remained infection-free for the past six months. She does not have significant dribbling at present.

## Discussion

Female hypospadias is defined as an abnormal ventral displacement of the urethral meatus along with foreshortening of the urethra. It is a relatively rare and poorly studied anomaly. The nomenclature is often used interchangeably with urogenital sinus, though the majority use the term to describe the urethral meatus that is located within 1.5cm from the introitus [1]. Solov'ev has classified the anomaly as partial (vestibular), subtotal (vestibulo-vaginal) and total (vaginal) [5]. Derevianko et al. have proposed another classification as low vaginal ectopia, high vaginal ectopia, urogenital fusion of the bladder neck with vagina and hypospadias associated with false or true hermaphroditism [2]. Sarin et al. have proposed using the term female hypospadias for very low anomalies where the urethra opens in the lower one-third of the vagina and urogenital sinus for situations where the urethral orifice opens in the upper two-thirds of the vagina. This has prognostic implications because the higher the urethral orifice is on the wall of the vagina, the narrower the urethra, and the more likely the chances of outflow obstruction and obstructive nephropathy [1].

The usual clinical presentations of children with hypospadiac urethra are dysuria and urgency, recurrent UTIs, urinary retention or dribbling. Children can also present with antenatally detected hydroureteronephrosis, or with elevated serum creatinine, or renal failure. The majority of patients are diagnosed while attempting a catheterisation. Female hypospadias is seen in association with 46,XX disorders of sexual development (DSD), urethral duplication, non-neurogenic neurogenic bladder, ectopic ureter and renal anomalies. Genital anomalies like septate vagina and bifid uterus can also occur in concurrence [3,4,6]. Many children with isolated hypospadiac urethra may remain asymptomatic and present later in their adolescence or adulthood with dyspareunia or recurrent lower urinary tract symptoms.

All five children described in our series had a normal-looking introitus and posterior fourchette with the urethral meatus identified on the anterior vaginal wall within the lower one-third of the vagina. They had early-onset recurrent UTIs. Among the patients, while four out of five of the children had increased frequency, one child had true urinary incontinence with a small capacity bladder. Urinary retention with abdominal voiding was present in three out of five children, of which, one child's symptoms improved after the excision of the dorsal urethral duplication cyst. Meatal stenosis was not present in any of these children, although the urethral wall was thin posteriorly, likely due to muscular deficiency. All of them had trabeculated bladder with the function of at least one kidney compromised. They also had poorly formed trigone. The symptoms of voiding dysfunction and cystoscopy findings of the children are strongly suggestive of neurogenic bladder. This may be associated with abnormal neuromuscular development of the bladder neck in these children. Four children had normal lumbosacral spine and one child had partial sacral agenesis with absent cord tethering on MRI (Table 1). The urodynamic study, however, was not done in any child due to technical difficulties.

**Table 1:** Patient presentation, investigations, management and follow-up UTI: urinary tract infection; RFT: renal function test; USS: ultrasound; R: right; L: left; B/L: bilateral; HUN: hydroureteronephrosis; PVR: post-void residual; MCU: micturating cystourethrogram; VUR: vesicoureteric reflux; DMSA: nuclear scintigraphy; RF: relative function; CIC: clean intermittent catheterisation; CMD: cortico-medullary differentiation * indicates the age at first presentation (in years).

Age^*^	Presentation	Examination findings	Associated anomalies	Cystogenitoscopy	Management and follow-up
3	Recurrent UTI, increased frequency, dribbling	Abdomen normal, RFT - normal, USS - R mild HUN, bladder PVR/volume - 20 ml/110 ml, MCU - R Grade 3 VUR, DMSA - R renal RF 35%, scar + MRI spine - normal	Nil	Urethral meatus 0.5 cm proximal to introitus, trabeculated bladder with flakes, patulous R ureteric orifice	Failed medical therapy, R ureteric re-implantation with Mitrofanoff, UTI free on CIC
2	Recurrent UTI, increased frequency, dribbling	Bladder palpable, S. Creatinine 2.5 mg/dL, USS - Gross HUN, bladder wall thickness 4 mm, PVR 64 ml, MCU - B/L Grade V VUR, DMSA - B/L renal scarring, MRI spine - normal	Anterior ectopic anus	Urethral meatus just proximal to the bladder neck, short urethra, open bladder neck, trabeculated bladder with B/L golf hole ureteric orifices, poorly formed trigone	B/L ureteric re-implantation with Mitrofanoff, S. Creatinine 0.5 mg/dL on CIC
3	Recurrent UTI, straining on micturition, urinary retention	Bladder distended to umbilicus, S. Creatinine 2.3 mg/dL, MCU - trabeculated bladder, L Grade V VUR, DMSA - L renal RF 25%, scars + MRI spine - normal	Nil	Urethral meatus 1 cm proximal to introitus, short urethra, poorly formed bladder neck and trigone, trabeculated bladder, patulous L orifice, R orifice mild dilatation	Continuous bladder drainage, being planned for L ureteric re-implantation with Mitrofanoff, S. Creatinine 0.8 mg/dL on continuous bladder drainage
7	Recurrent UTI, urinary incontinence	Abdomen normal, RFT - normal, USS - R mild HUN, LK contracted, MCU - small capacity bladder, R Grade III VUR, DMSA - L renal RF 2%, MRI - short urethra, incomplete septate vagina, bicornuate bicollis, spine - normal	Bicornuate bicollis with rudimentary uterus and cervix	Urethral meatus 1 cm proximal to introitus, short urethra, small capacity bladder, ill-defined trigone. R orifice patulous, laterally placed, L orifice non-visualized, oblique vaginal septation with cervix in the posterior part	Being planned for bladder augmentation with Mitrofanoff, S. Creatinine 2.3 mg/dL at present, R kidney CMD lost on follow-up USS
1	Recurrent UTI, straining on micturition, urinary retention	Abdomen - normal, clitoromegaly + USS - L HUN with paraureteric diverticulum, MCU - L diverticulum, no VUR, DMSA - L renal RF 15%, scars + MRI spine - partial sacral agenesis, no tethering	Anterior ectopic anus, clitoromegaly, aborted dorsal urethral duplication	Cystic dorsal urethra within corpus spongiosum, short ventral urethral opening 1 cm proximal to introitus, short urethra, trabeculated bladder with large left paraureteric diverticulum	Excision of dorsal cystic urethra, anterior sagittal anorectoplasty, left nephroureterectomy, UTI-free for six months

Association of anterior ectopic anus in 40% (2/5) and genital anomaly in 20% (1/5) of our patients suggests abnormal urogenital sinus development. Twenty per cent (1/5) had a spinal abnormality, which would have contributed to the neurogenic bladder. The need for complete bladder emptying in these children cannot be overemphasized [3,7]. The two children who underwent ureteric reimplantation with Mitrofanoff for CIC improved symptomatically in terms of UTI and voiding frequency. While one previous case report documented a higher risk of stricture when urethral reconstruction is attempted at an early age, another report has suggested attempting urethral reconstruction for improvement of voiding symptoms or for CIC.

Varying techniques of correction of the anomaly have been described, all aimed at reconstruction of the urethral opening to just below the clitoris. These techniques include meatoplasty, urethral meatal transposition [8-10], tubularisation of the vaginal wall and covering it with a pedicled flap of labial or bulbocavernous muscle [1], reconstruction of the urethra using the vaginal wall along with a perineal or gluteal flap, and a tubularisation of a bladder flap [11].

## Conclusions

Female hypospadias presenting as recurrent UTIs in children has a strong association with non-neurogenic neurogenic bladder. Unlike patients who first present in their adolescence, children with female hypospadias have associated voiding dysfunction, which has to be carefully evaluated. There is a high risk of renal failure in these children from an already compromised upper tract and recurring infections. Early intervention for bladder drainage is essential to prevent progressive upper urinary tract damage. Primary urethral reconstruction alone may not resolve their symptoms or change the disease course.
